# Multiscale Sensing of Bone-Implant Loosening for Multifunctional Smart Bone Implants: Using Capacitive Technologies for Precision Controllability

**DOI:** 10.3390/s22072531

**Published:** 2022-03-25

**Authors:** Inês Peres, Pedro Rolo, Jorge A. F. Ferreira, Susana C. Pinto, Paula A. A. P. Marques, António Ramos, Marco P. Soares dos Santos

**Affiliations:** 1 Department of Mechanical Engineering, University of Aveiro, 3810-193 Aveiro, Portugal; inesperes@ua.pt (I.P.); pmrolo@ua.pt (P.R.); jaff@ua.pt (J.A.F.F.); scpinto@ua.pt (S.C.P.); paulam@ua.pt (P.A.A.P.M.); a.ramos@ua.pt (A.R.); 2 Centre for Mechanical Technology & Automation (TEMA), University of Aveiro, 3810-193 Aveiro, Portugal

**Keywords:** smart implants, instrumented medical device, implant technology, bioelectronic implants, capacitive sensing, computing in medical devices

## Abstract

The world population growth and average life expectancy rise have increased the number of people suffering from non-communicable diseases, namely osteoarthritis, a disorder that causes a significant increase in the years lived with disability. Many people who suffer from osteoarthritis undergo replacement surgery. Despite the relatively high success rate, around 10% of patients require revision surgeries, mostly because existing implant technologies lack sensing devices capable of monitoring the bone–implant interface. Among the several monitoring methodologies already proposed as substitutes for traditional imaging methods, cosurface capacitive sensing systems hold the potential to monitor the bone–implant fixation states, a mandatory capability for long-term implant survival. A multifaceted study is offered here, which covers research on the following points: (1) the ability of a cosurface capacitor network to effectively monitor bone loosening in extended peri-implant regions and according to different stimulation frequencies; (2) the ability of these capacitive architectures to provide effective sensing in interfaces with hydroxyapatite-based layers; (3) the ability to control the operation of cosurface capacitive networks using extracorporeal informatic systems. In vitro tests were performed using a web-based network sensor composed of striped and interdigitated capacitive sensors. Hydroxyapatite-based layers have a minor effect on determining the fixation states; the effective operation of a sensor network-based solution communicating through a web server hosted on Raspberry Pi was shown. Previous studies highlight the inability of current bone–implant fixation monitoring methods to significantly reduce the number of revision surgeries, as well as promising results of capacitive sensing systems to monitor micro-scale and macro-scale bone–interface states. In this study, we found that extracorporeal informatic systems enable continuous patient monitoring using cosurface capacitive networks with or without hydroxyapatite-based layers. Findings presented here represent significant advancements toward the design of future multifunctional smart implants.

## 1. Introduction

**Figure 1:**
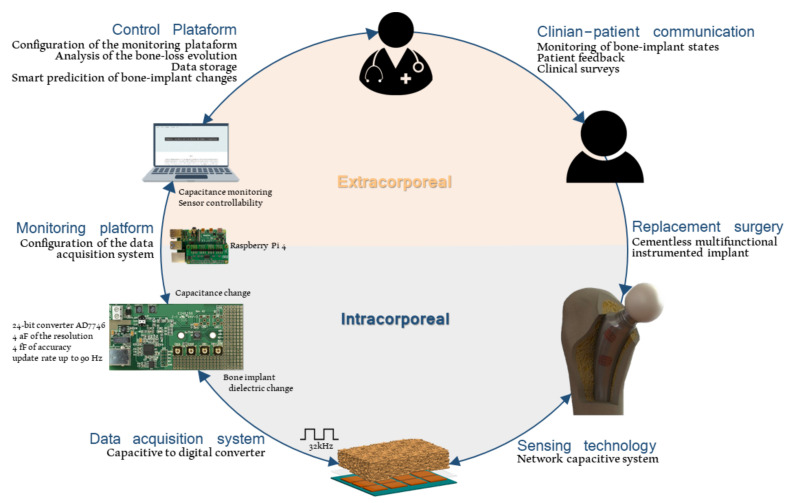
Schematic overview of a bioelectronic implant. The information flow is also included to highlight the data transfer between the implant and extracorporeal systems that allow clinicians to analyze bone–implant interface states from a distance.

Population growth and extended life span have increased the prevalence of non-communicable diseases, namely those related to musculoskeletal disorders [1,2]. These are responsible for more than 20% of the number of years lived with disability [1]. Included is osteoarthritis, currently accounting for 4% of global prevalence [1]. Around 70% of knee replacements and 27% of hip replacements are due to obesity-driven osteoarthritis [3], although age, sex, trauma and joint morphology are also relevant patient-specific risk factors [4]. Knee and hip arthroplasties are among the most frequent surgeries performed worldwide, having a relatively high success rate nowadays [4]. Nonetheless, around 7% of patients need to undergo revision surgery after five years and 12% after ten years [5]. Moreover, 15–20% of patients still suffer from ongoing pain and poor function [1,6]. The risk of revision surgery is about 10%, but substantially higher among younger patients (60% is predicted by 2030) [6,7]. The increase in young patients undergoing bone replacement procedures increased the usage of cementless fixations [8]. This type of fixation requires a high bone–implant interaction, both at nano and micro level scales, to guarantee effective osseointegrations. However, bone density decrease, low modeling/remodeling rates, and stress-shielding can result in aseptic loosening and, ultimately, in implant failure [4,7,9,10]. To prevent alarming revision surgery rates, effective osseointegrations must be ensured. Indeed, peri-implant loosening can exceed 50% of revision procedures [4,6]. Bone–implant fixation can be enhanced by coating implants with hydroxyapatite, as 40% of the bone structure is composed of minerals (plus 35% of collagen and 25% of water) [11], which is predominantly composed of calcium and phosphate, arranged similarly to synthetic hydroxyapatite crystals [12]. This technique reduces the body’s rejection risk to the inserted implant and the number of implant failures [13]. Furthermore, many research efforts have been conducted to develop more sophisticated bioactive biomaterials for coating uncemented implants [14,15]. Even so, these methodologies will not conduct optimal bone–implant fixation states, mainly due to their inability to consider the bone–implant fixation state to personalize bioactivity [16,17,18]. So, an effective monitoring of the bone–implant fixation is required to minimize revision surgeries. Currently, the bone–implant fixation monitoring and the decision for revision surgery is based on imaging methods. These methods have low accuracy in detecting early loosening stages and cannot be performed throughout the daily life of patients. By consequence, they cannot establish high-performance osteoinduction and osteoconduction processes according to personalized requirements [18,19]. Instrumented multifunctional implants hold the potential to monitor the bone–implant interface and deliver personalized stimulation to peri-implant tissues [7,20,21]. By incorporating electronics within the implant, research studies have focused on collecting different biomechanical quantities, such as forces, temperatures and moments of inertia, and more recently, detecting bone loss stages using the following methodologies [19]: acoustic [22,23,24,25,26,27], vibrometric [23,26,28,29,30,31,32,33,34], magnetic induction [28,35], deformation [36,37] and bioelectric impedance [38,39]. Although developed technologies in this scope provide significant advances relative to imaging methodologies, they are not able to perform effective monitoring of different loosening stages. Indeed, the following problems still need to be addressed [19]: (i) provide flexible integration to sensing systems inside the instrumented implants; (ii) provide an easy way to redesign the detection technologies for different geometries of implant surfaces; (iii) provide target-oriented monitoring of peri-implant regions; (iv) provide continuous monitoring of patients during their quotidian life. Soares dos Santos et al. [7,18,20] recently proposed cosurface capacitive technologies both to deliver electrical stimuli to bone structures and to monitor bone–implant fixation states. They found promising in vitro results to enhance osteoconduction and to detect both macro-scale and micro-scale biointerface bone loss. Furthermore, these capacitive systems are low cost and non-complex architectures, well-suited for multiple implant surfaces and with the ability to perform stimulating-sensing operations in target bone regions [18,20]. Therefore, they demonstrated the ability to overcome the limitations of the previously proposed sensing methodologies. Although these capacitive sensing systems have already provided successful results concerning monitoring loosening stages, it is currently unknown if they are able to operate: (1) as a sensing network-based solution, as required by smart multifunctional instrumented implants; and (2) as effective sensing systems in biointerfaces incorporating hydroxyapatite-based layers, as required by current implant technologies in the market. The findings obtained here highlight success in both scenarios. Moreover, the influence of the stimulation frequency on the sensor sensibility was analyzed, which is mandatory for this capacitive system to operate both as a sensor and stimulation system. Figure 1 shows a schematic of the proposed sensing system comprising extracorporeal and intracorporeal parts. Both patients and clinicians/surgeons are able to extracorporeally follow up the bone–implant fixation states after implant insertion, as bioelectronic implants intracorporeally perform personalized sensing operations, data acquisition and remote communication. Therefore, this study intends to validate the concept of cosurface monitoring sensors in implants and provides a significant contribution to the development of future multifunctional smart implants.

## 2. Materials and Methods

### 2.1. Capacitive Sensors

**Figure 2:**
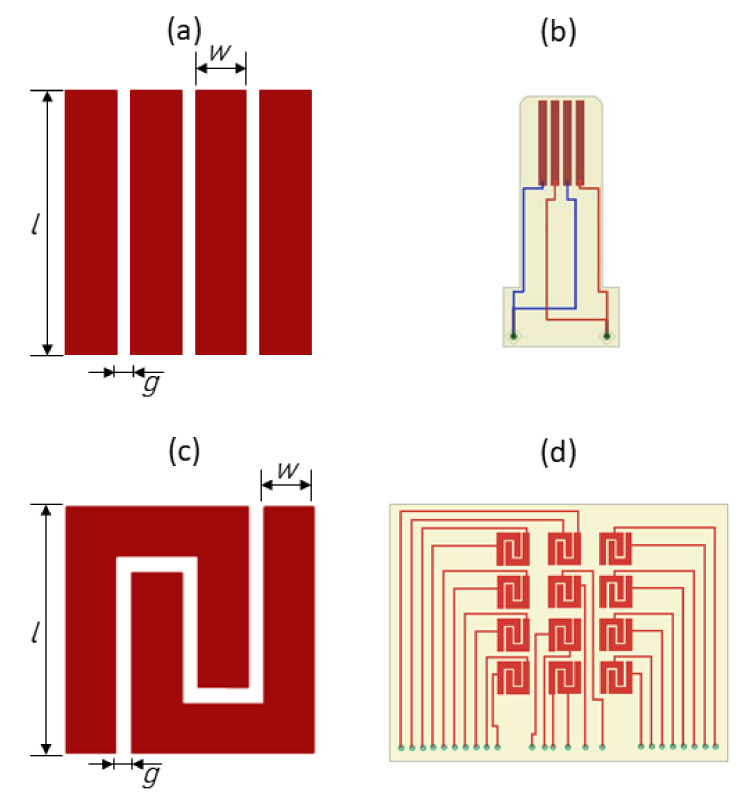
Capacitive architectures: (**a**) Striped sensor design (l = 10 mm; w = 1 mm; g = 0.5 mm); (**b**) Electrical schematic of the striped cosurface capacitive sensor; (**c**) Interdigitated sensor design (l = 10 mm; w = 1 mm; g = 0.5 mm); (**d**) Electrical schematic of the cosurface capacitive network.

Striped and interdigitated cosurface capacitive sensors were engineered, as shown in Figure 2. The striped architecture, very similar to the one developed by Soares dos Santos et al. [7], comprises four electrodes spaced by 0.5 mm *(g)*, having 10 mm length *(l)*, 1 mm thickness *(w)*, and a total footprint of 10×5.5×0.1 mm (Figure 2a). A single architecture was used to test the influence of the hydroxyapatite-based layer on the sensing performance (Figure 2b). The interdigitated architecture was designed with the same dimensions used for the striped architecture (Figure 2c). Twelve interdigitated capacitors were organized on a 4×3 matrix architecture, comprising the following features: 2.5 mm distance between rows and 4 mm distance between columns, 0.5 mm gap *(g)* between electrodes (Figure 2c,d). Electrodes of the capacitive sensors were manufactured with copper due to its high electrical conductivity and reduced magnetic properties. All capacitive systems were fabricated using Printed Circuit Board (PCB) technology with FR4 substrate.

### 2.2. Monitoring Apparatus for Web-Based Networked Sensing

**Figure 3:**
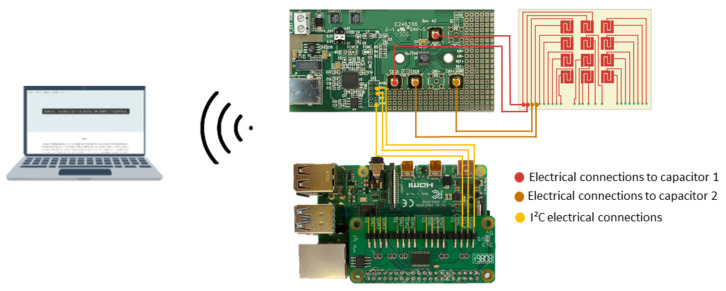
Schematic of the overall Master Active Structure.

In the previous study performed by Soares dos Santos et al. [7], the feasibility of using a capacitive sensing system to monitor different loosening states was proven. The present study used a network of capacitors to evaluate the bonding states in a larger area of the bone–implant interface. Electrodes were powered with 32 kHz of excitation frequency (LF test), the same frequency excitations used in the above-mentioned test [7]. A Raspberry Pi was programmed as a Master structure with the ability to control the sensing operation of bioelectronic implants, now defined as a slave system, such that personalized bone–implant fixation states can be extracorporeally monitored (by a control system). This overall methodological design, based on an intracorporeal sensing system controlled by an extracorporeal structure, is required to optimize the implant performance (indeed Soares dos Santos et al. [17] provided an optimality analysis on orthopaedic implants, in which they demonstrated that optimal implant performances require some kind of sensing, actuation, communication and self-powering systems). Previous works only considered local data acquisition [40,41]. We presented an innovative sensing approach in which the Master platform was implemented comprising (Figure 3): (i) a PCB-based capacitive network; (ii) capacitance-to-digital converters; (iii) an I${}^{2}$C multiplexer; (iv) a Raspberry Pi. The capacitance-to-digital converter and capacitive network were electrically connected through an RG174 coaxial cable. The chosen capacitance-to-digital converter was the same as that used by Soares dos Santos et al. [7]: 24-bit converter AD7746 (Analog Devices, Norwood, MA, EUA), which provides 4 aF ($4\times 10^{-18}$ F) of the resolution, 4 fF ($4\times 10^{-15}$ F) of accuracy, 0.01% of linearity, ±4 pF ($4\times 10^{-12}$ F) of full-scale changing capacitance range, and update rate up to 90 Hz.

**Figure 4:**
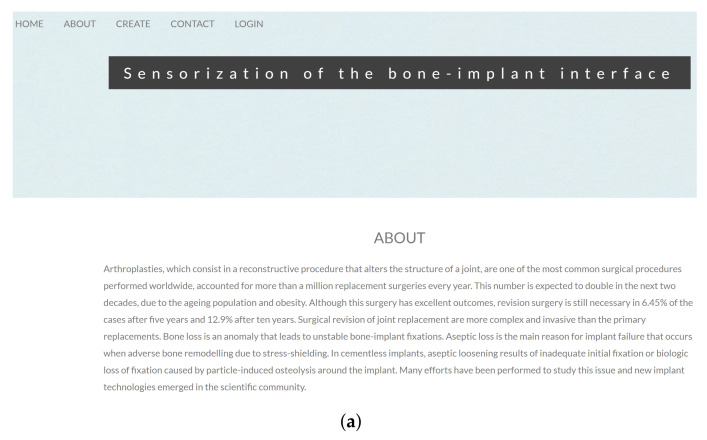

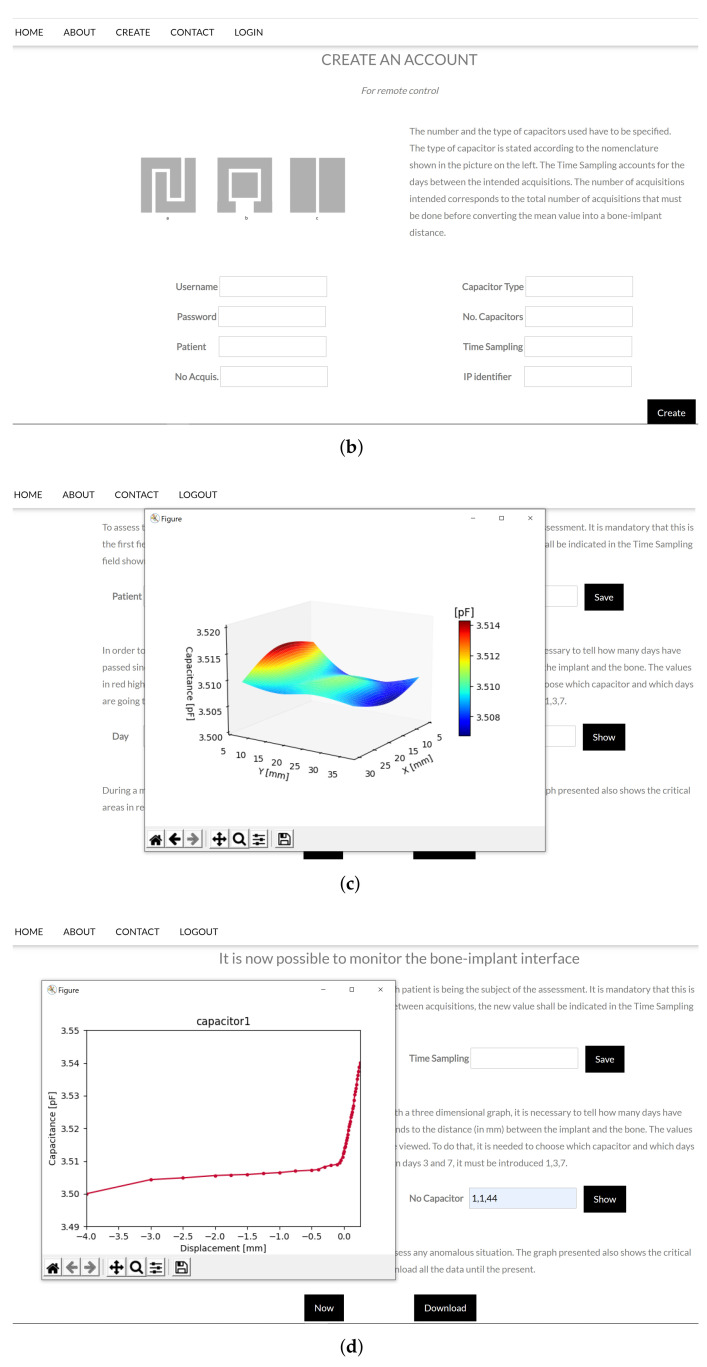
Developed website: (**a**) home page; (**b**) form to create an account; (**c**) control page with a tridimensional plot representing the bone–implant fixation state monitored by a capacitive sensing network for multiple regions; (**d**) control page with a bidimensional plot representing the bone–implant fixation state monitored by a capacitive sensing network for a specific region (detected by a specific sensor of the network).

Six converters were used as each one only allows data acquisition from two capacitors. All AD7746 have the same I${}^{2}$C slave address, prohibiting the direct connection between the board and the reading system. An I${}^{2}$C multiplexer enables the selection of a specific channel before indicating the converter’s slave address. By using an I${}^{2}$C multiplexer, it was possible to connect all the AD7746 converters to a Raspberry Pi, which was chosen as the high-level device of this acquisition system, once it allows simultaneous I${}^{2}$C communication with converters and the control system. To enable communications between the Master platform and the control platform, the Raspberry Pi was turned into a web server by using APACHE, PHP, and MySQL frameworks. Capacitance values were acquired by algorithms coded in PYTHON, with a specific time sampling defined by the control system, and stored on the database. The control platform required: a hardware part, consisting of a computer converted into a web server by using DJANGO framework; and a software part providing a web application. The developed web application (Figure 4a) enables the configuration of the Master system.

**Figure 5:**
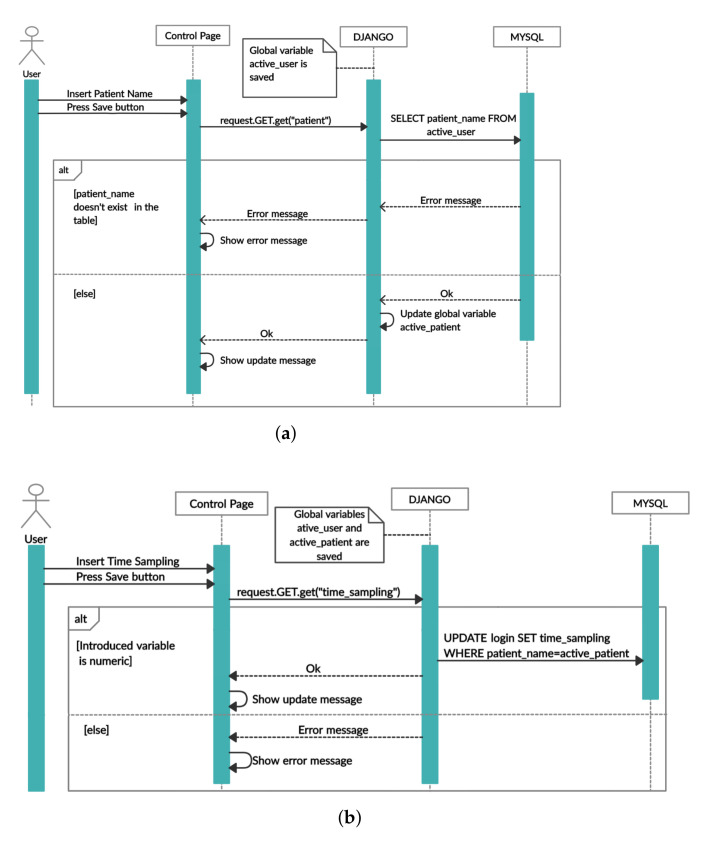
UML sequence: (**a**) to update patient record; (**b**) update time sampling.

**Figure 6:**
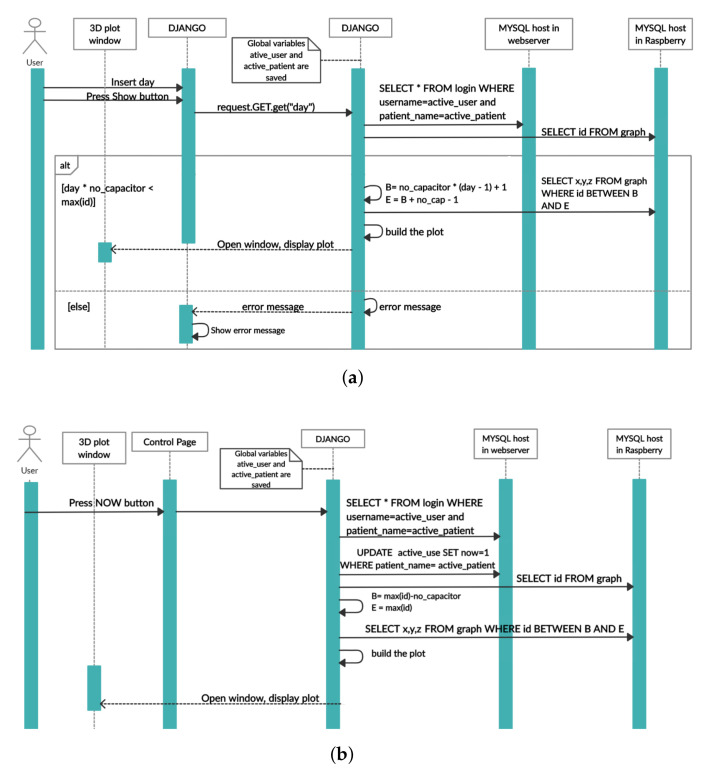
UML sequence (**a**) to display the three dimensional plot; (**b**) to display the current capacitance values.

A successful configuration demand to create an account with the following information (Figure 4b): (i) identification for login (username and password); (ii) patient name; (iii) total number of acquisitions that the Master must perform before storing the mean value into the database; (iv) capacitive architecture; (v) number of capacitors in the network; (vi) time sampling that the Master application must wait between acquisition; (vii) and Raspberry Pi IP. Inserted information is recovered in a localhost database. After an account is created, due to security reasons, the user has to log in to proceed to the patient implant analysis on the control page. Before proceeding to analyses focused on bone–implant fixation states, it is necessary to insert the patient name so that the Master extracorporeal system and the Raspberry Pi hosted in the slave-designed implant can be connected (Figure 5a). This step allows the analysis of the acquired data in different plottings: (i) Tridimensional representation of the region-dependent capacitance related to bone–implant fixation states (Figure 4c); (ii) Bidimensional representation of the capacitance related to bone–implant fixation state changes in specific regions along the orthogonal axis (to the capacitive sensors), in which each region is associated to a single capacitor of the sensing network (Figure 4d). The acquired data can also be downloaded to Excel files. The software in the Master was designed to allow the updating of the patient record (Figure 5a) and customizing the time sampling (Figure 5b), according to the sequence of events highlighted in the Unified Modeling Language (UML) diagrams illustrated in Figure 5. As different dates may be associated with different bone–implant fixation states, the clinician must indicate the date to obtain tridimensional plottings (the first day corresponds to the surgery day). After filling the form, the connection to the Raspberry Pi is established, triggering the corresponding data to be read. Figure 6a represents the sequence UML of this task. Clinicians can also perform data monitoring in real-time (e.g., throughout medical appointments), as shown in Figure 6b.

### 2.3. Monitoring Apparatus of the Sensing System Using Hydroxyapatite-Based Layer

**Figure 7:**
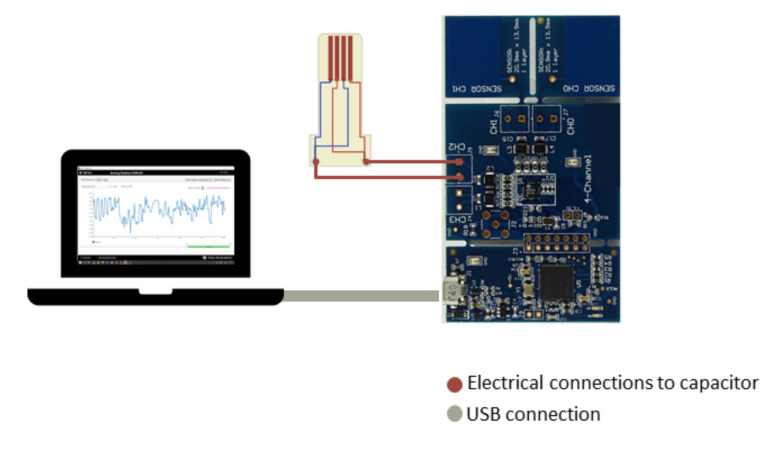
Schematic of the overall data acquisition system to perform HF tests.

The impact of using a hydroxyapatite-based layer between the capacitor (similar to the one used in [7]) and bone was analyzed using excitation frequencies of 32 kHz (LF test) and 4.8 MHz (HF tests). LF tests were performed using the same monitoring apparatus described by Soares dos Santos et al. [7] during the analysis with the striped architecture. As the previously described hardware (Section 2.2) did not allow significantly higher frequency excitations, HF tests were carried out using an FDC2212 EMI-Resistant 28-bit Capacitance-to-Digital Converter board (Texas Instruments). This board provides a 28-bit resolution, a frequency excitation range up to 10 MHz, and a maximum update rate of 245 Hz. To configure and control this conditioning circuitry, the EVM GUI software was used. Experimental tests were performed having a data acquisition rate of 50 Hz. Electrical connection between the data acquisition board and the capacitive system was established through RG174 coaxial cable and according to the scheme indicated in Figure 7.

### 2.4. Experimental Setup

**Figure 8:**
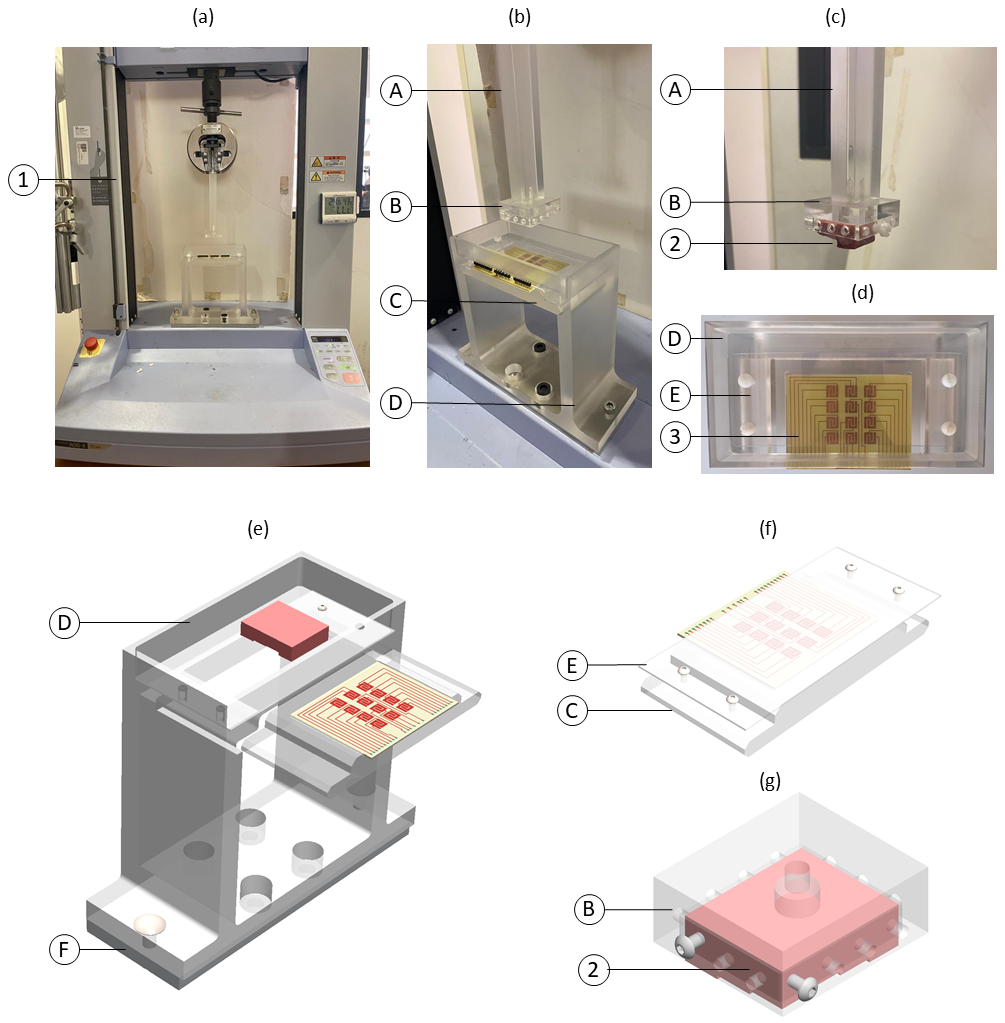
Experimental apparatus employed during in vitro tests: (**a**) Overall experimental apparatus; (**b**) Detailed view of the experimental apparatus: A—polymeric stem to connect the test machine and the hollow structure B to the bone samples (2); B—hollow structure to attach the network of twelve bone samples; C—polymeric structure to embed the capacitive network; D—polymeric hollow structure to attach capacitors and interfacial sheet; (**c**) Detailed view of structures A and B with a bone sample; (**d**) Detailed schematic of structure C; E—polymetric layer to cover the PCB capacitive architectures; (**e**) Schematic of the overall apparatus including structure F connecting the support platform to the testing machine; (**f**) Detailed schematic of structures C and E (**g**) hollow structure attached to the network of twelve bone samples. Legend: 1—testing machine; 2—bone samples; 3—network of capacitors.

To emulate the loosening process occurring after bone replacement surgeries, a similar experimental apparatus to the one proposed by Soares dos Santos et al. [7] was developed (Figure 8). Briefly, the experimental apparatuses only differ by the dimension of the hollow structures B and C. The bone–sensor contact was prevented by placing a polymeric sheet (structure E) with 1 mm thickness between these components. Apart from avoiding contact, the polymeric layer ensures a high electrical resistivity, which is required for electric stimuli delivery along the bone structures. During tests using hydroxyapatite-based layers, the bone-contact side of the polymeric structure was coated by this mineral-organic matrix alloy. Bone samples (structure 2) were vertically moved downward towards the capacitive structure using a universal machine (structure 1). Structure (A) was developed to establish required bone-coating distances. At the bottom extremity of structure (A), a hollowed structure (B) was positioned to incorporate the bone samples. Two apparatuses were engineered: one for the tests involving a hydroxyapatite-based layer (hollowed B structure with 15×15×7.5 mm${}^{3}$); and another for testing the capacitive networking (hollowed B structure with 32×39×7.5 mm${}^{3}$). Capacitive sensors were embedded in the motionless structure D connected to the bottom of the testing machine (structure F). PCB capacitive architectures were housed in a 2 mm depth structure (C). All screws and support components were manufactured in acrylic to avoid magnetic influences during the experimental tests.

### 2.5. Bone Samples

**Figure 9:**
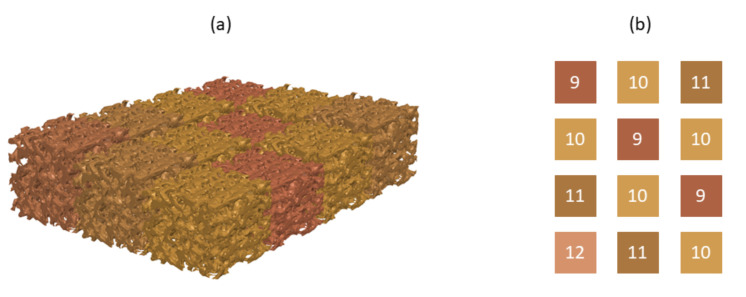
Bone assembly with a pattern of different heights for testing the capacitive sensing network: (**a**) Three-dimensional schematic of the bone assembly comprising twelve bone samples with different heights; (**b**) Height of each bone sample. The color correspondence also highlight the sensor position within the sensing network.

Experimental tests were conducted using trabecular samples of porcine femoral bone. Bone samples were machined according to two different shapes: (i) 24 cubic samples were manufactured (12 for LF tests and twelve for HF tests) with 10×10×10 mm${}^{3}$ for in vitro testing with hydroxyapatite-based layer; (ii) tests with the network capacitive system required 72 cuboid-shaped samples, which were manufactured with the same bidimensional measures (9.5×10.5 mm${}^{2}$) and different heights: 12 mm, 11 mm, 10 mm, and 9 mm, in a total of 6, 18, 30, and 18 samples, respectively. Each analysis used a total of 12 bone samples with different heights, as illustrated in Figure 9. This bone assembly was designed to test the ability of the sensing system to detect both loosening states and fixation states.

### 2.6. Hydroxyapatite Layer Characterization

**Figure 10:**
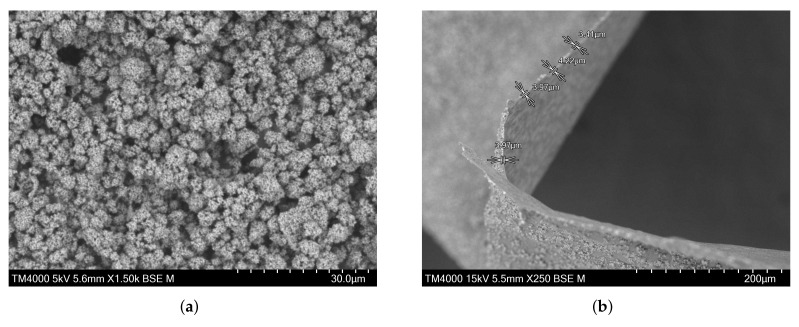
Scanning electron micrographs of: (**a**) hydroxyapatite-based layer surface and (**b**) detached layer from the polymeric plate.

A hydroxyapatite-based layer was used to mimic implant–bone interfaces resulting from uncemented implants comprising (worldwide used) hydroxyapatite coatings [42]. The preparation of this mineral-organic matrix component was performed considering the composition of human bone [12,43]. The gelatin (denatured collagen) was selected as a binder of the hydroxyapatite particles due to its adhesiveness and plasticity [44]. A non-homogeneous and porous layer was obtained, as shown in Figure 10, in which the average of the overall coating thickness has 3.89 μm, as collagen is found structurally integrated within mineral agglomerates for such a thickness [45].

### 2.7. Preparation of the Hydroxyapatite-Based Layer

The experimental procedure to obtain the final hydroxyapatite layer was carried out as before [46]. Briefly, it consisted of dissolving 1 g of gelatin (from porcine skin) in 10 mL of distilled water using a magnetic stirrer at 300 rpm and with heating for 10 min at 40 ${}^{\circ }$C. After, 2 g of hydroxyapatite (CAPTAL^®^’R’ sintering grade) were added to the gelatin solution and stirred for more 15 min. Following that, 10 drops of the suspension were placed onto an acrylic layer (same to those used in the tests without the layer) to make a thin coating, which was then allowed to cure for 48 h at room temperature. After drying, an analysis was performed using the Scanning Electron Microscope scanning (SEM) (TM4000Plus, Hitachi, Tokyo, Japan).

### 2.8. Experimental Procedure for *In Vitro* Tests

**Figure 11:**
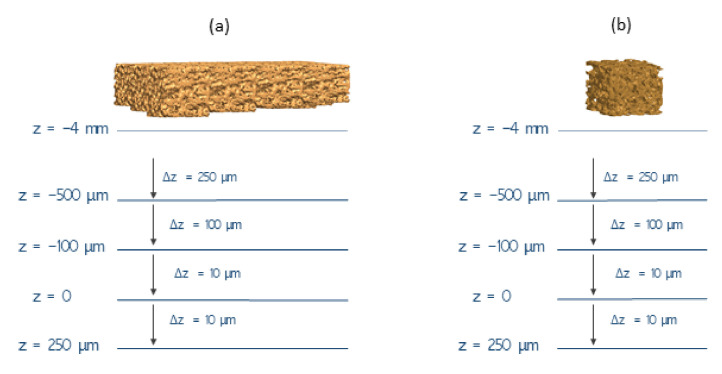
Experimental procedure to mimic different implant–bone interfaces: (**a**) using a capacitive sensing network; (**b**) using a single capacitive sensor.

To simulate different bone–implant fixation states, experimental tests were carried out by analysing capacitance variations during bone approximation to the sensing platform. These tests were conducted along a vertical downward trajectory from a state of severe bone loss (scenario defined by bone–sensing interface contactless) to a state of bonding (scenario defined by the contact with the polymeric layer). The contact between bone specimens and the polymeric layer was defined as the position z = 0. Bone loosening states were defined as negative positional values; bonding was defined as positive values. All experimental tests were performed using the same loosening and bonding states, which correspond to the same positional protocol (Figure 11) in which capacitances were measured. The downward trajectory started at point z = −4 mm (severe loosening state), and steps of 250 μm were made up until the z = −500 μm position was reached (a loosening state). The displacement steps were then reduced to 100 μm to z = 100 μm. Displacements were even shortened to 10 μm until very weak bonding occurs (z = 0), as well as during the last stage towards a strong bonding scenario (up to z = 250 μm). Notice that throughout analyzing the capacitive network performance, as bone samples have different heights, only the bone sample with a 12 mm height reached the bone–sensing interface contact; the other samples (11, 10 and 9 mm of height) remain in loosening scenarios (distances to the bone–sensing interface: −1, −2, and −3 mm, respectively). The experimental tests were conducted using the Trapezium X (Shimadzu) testing machine (Figure 8, structure 1). The developed support (Figure 8) was fixed at its bottom by an aluminum board. All the performed tests were under the following environmental conditions: 26–28 ${}^{\circ }$C and 50–55% humidity.

## 3. Results

### 3.1. Experimental Performance of the Networked Sensing System

**Figure 12:**
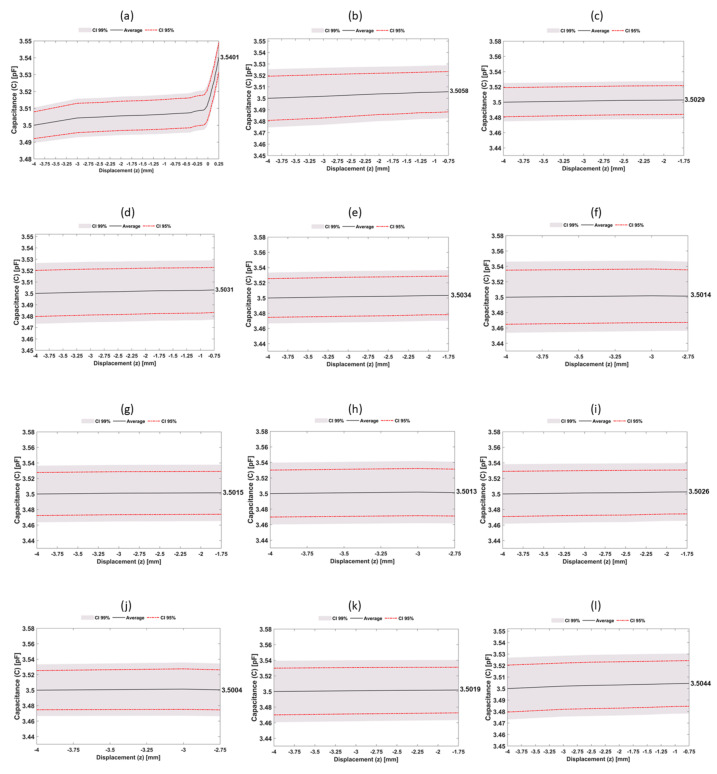
Experimental results of in vitro tests following a vertical downward trajectory from −4 mm to 250 μm (corresponding to a trajectory from severe loosening to a strong bonding) using a capacitive sensing system and the bone assembly with a pattern of different heights: (**a**) results obtained for a bone sample with 12 mm height; (**b**,**d**,**l**) results obtained for a bone sample with 11 mm height; (**c**,**e**,**g**,**i**,**k**) results obtained for a bone sample with 10 mm height; (**f**,**h**,**j**) results obtained for a bone sample with 9 mm height.

All experimental tests showed a similar variation trend in capacitance measurements: a highly non-linear capacitance increase is observed as the bone sample approaches the capacitive sensing system (Figure 12). Regardless of the capacitor architecture and excitation frequency, significant capacitive increase rates mainly occur for bonding states (bone–sensing interface contact scenarios). Bone samples with a 12 mm height were the only ones that allowed performing the overall trajectory from severe bone loss state to the strong bonding state (−4≤z≤0.25 mm) (Figure 12a). The average capacitance variation was 40.1 fF (9.4fF/mm), in which about 68% of this variation occurred during the bonding states (0≤z≤250μm).

Concerning 11 mm height bone samples (Figure 12b,d,l), the performed trajectory was from severe bone loss to a loosening state (−4≤z≤−0.75 mm).

The average capacitance variation was 4.43 fF (1.38fF/mm), representing an 85% reduction in the capacitance change per each millimetre compared to the 12 mm bone samples.

**Figure 13:**
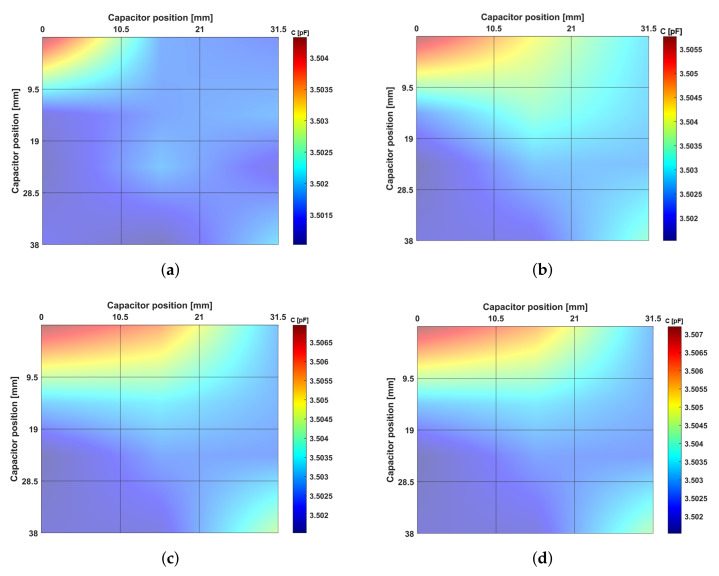
Three-dimensional representation of the variation of the electrical capacitance measured by the capacitive sensing network during in vitro tests: (**a**) 12 mm height bone samples for a severe loosening (z = −3 mm); (**b**) 12 mm height bone samples for a loosening state (z = 500 μm); (**c**) 12 mm height bone samples for a very week bonding state (z = 0); (**d**) 12 mm height bone samples for a strong bonding (z = 250 μm).

This sensibility decrease occurs because the capacitive sensors present a much higher sensibility detecting small-scale debonding disorders (0≤z≤250μm), as observed in Figure 12a. The 10 mm height bone samples (Figure 12c,e,g,i) also performed trajectories between two severe bone losses (−4≤z≤−1.75 mm). The average capacitance variation was 2.46 fF (1.104fF/mm), corresponding to decreasing rates of the average capacitance per each millimetre of 20% for the 11 mm height samples and 88% for the 12 mm height samples. For the smallest set of samples (9 mm, Figure 12f,h,j), trajectories from two different severe bone loss states (−4≤z≤−2.75 mm), resulted in the lower average capacitance variation, namely 1.21 fF (0.94fF/mm). The capacitance variation per each millimetre obtained in these samples was 14%, 31%, and 89% lower than that obtained for the 10, 11 and 12 mm samples, respectively. Results obtained using the 12 mm height bone samples showed that higher capacitance variations occurred in the range from weak to strong bonding scenarios, which demonstrate the ability of this network sensing system to detect early loosening states. Indeed, early loosening states (decreasing bone–sensor contact, corresponding to trajectories approaching z = 0) are the most relevant ones to prevent implant failures: the stimuli delivered by smart implants must prevent loosening scenarios (no bone–sensor contact), hence preventive therapeutic stimulation must be mainly focused throughout trajectories from strong to week bonding (0≤z≤250μm). Nevertheless, although (de)bonding scenarios present higher detecting sensibility relative to loosening scenarios, these analyses also highlight the ability of the network sensing system to detect different loosening states. Notice that it is quite clear what regions are loosed and those which are in a fixation state, by observing the last *x*-axis values of all sub-figures of Figure 12. Therefore, therapeutic stimulation can also be delivered to bone–implant interfaces with severe loosening states, such that trajectories from failure states to no-failure states can be successfully performed. Figure 13 highlights the detection ability of the network sensing system: extended peri-implant regions (along the sensing surface and orthogonal directions) can be monitored; three-dimensional plots allow to identify the overall fixation scenario, in which both bonding and loosening states can be easily observed. As higher capacitance values are related to strong bonding scenarios, loosening scenarios are easily detected if near bonding states are also detected, since significant capacitance differences are monitored.

### 3.2. Experimental Performance of the Sensing System with Hydroxyapatite-Based Layer

**Figure 14:**
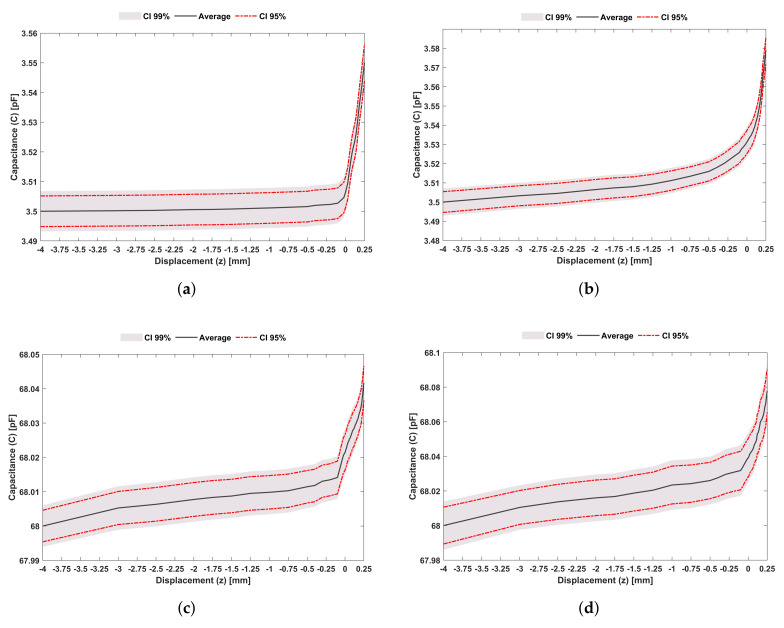
Experimental results of the sensing system with hydroxyapatite-based layer following a vertical downward trajectory from −4 mm to 250 μm (corresponding to a trajectory from severe loosening to a strong bonding) using the striped co-surface architecture: (**a**) LF tests without the hydroxyapatite-based layer between the bone sample ad the capacitive structure; (**b**) LF tests including the hydroxyapatite-based layer in the same biointerface; (**c**) HF tests without the hydroxyapatite-based layer between the bone sample ad the capacitive structure; (**d**) HF tests including the hydroxyapatite-based layer in the same biointerface.

During LF tests (Figure 14a,b), the average capacitance variation from severe loosening to strong bonding (−4≤z≤0.25 mm) was 50 fF (1.18fF/mm) without the hydroxyapatite-based layer (Figure 14a) and 79.18 fF (1.86fF/mm) with the hydroxyapatite-based layer (Figure 14b). Thus, a 58% increase in the average capacitance is found when this mineral-organic layer is inserted between the bone and the sensing interface. During bonding states (0≤x≤250μm) and without hydroxyapatite-based layer, the average capacitance variation was 43.7 fF; introducing the mineral-organic layer, 47.9 fF capacitance changes were observed, representing an increase of about 9.5%. Concerning HF tests (Figure 14c,d), the average capacitance variation was 41.7 fF (9.8fF/mm) without hydroxyapatite-based layer (Figure 14c) and 77.80 fF (18.3fF/mm) with hydroxyapatite-based layer (Figure 14d), i.e., an increase of 86% with the use of the mineral-organic layer. Throughout trajectories from weak bonding to strong bonding (0≤x≤250μm), the average capacitance variations were 20.3 fF without hydroxyapatite-based layer and 38.7 fF with hydroxyapatite-based layer.

Hence, without any mineral-organic layer, capacitance changes during bonding states were about 50% of the average capacitive variation monitored from severe loosening and strong bonding; however, these capacitive changes were significantly higher when the hydroxyapatite-based layer was used: changes of about 90% higher were detected. Regardless of the experimental test and the usage of hydroxyapatite-based layer, sensing sensibility increased for interfaces comprising a mineral-organic layer. Indeed, considerable capacitive differences were obtained from loosening states to bonding states (35 pF for LF tests and 68 pF for HF tests in the range −4≤z≤0.25 mm). Without the layer, the average capacitance variation was 20% higher for LF tests (9.8 fF for HF tests and 11.8 fF for LF tests). During the bonding states (0≤z≤250 µm), higher average capacitance variations were observed during LF tests (54% reduction in HF tests). While using the layer, the mean capacitance variation was 79.2 fF (1.86 fF/mm) for LF tests and 77.8 fF (1.83fF/mm) for HF tests, which corresponds to a 2% reduction in HF tests. These results (high sensing sensibility for LF tests) are very relevant, as very recent research found that the delivery of cosurface capacitive-driven electric stimuli around the LF excitation frequency is highly effective at promoting osteoconduction and osteoinduction [18]. Therefore, the same cosurface capacitive network sensing can effectively operate as an acting-sensing system even for interfaces comprising a mineral-organic layer. The experimental performance of the capacitive sensing network using interfaces with hydroxyapatite-based layer was also evaluated. Similar results to the ones presented in Figure 12 were achieved, although with the capacitive changes observed in Figure 14.

## 4. Discussion

This study provides additional evidence that the next technological revolution in the field of implantable bone devices will most likely occur with the design of multifunctional smart implants. The use of a network of capacitors was able to efficiently detect different bone–implant fixation states (from severe loosening to strong bonding) in a larger peri-implant area (38×32 mm) than the previous works already conducted in this scope [7] (up to 10×10 mm). Indeed, the capacitive sensing network allowed simultaneous analysis of different bone–implant fixation states during in vitro tests using a bone assembly with a pattern of different heights (Figure 12 and Figure 13). Higher detection sensibility was found from weak fixations to strong bonding states. Therefore, capacitive systems provide a better performance in detecting early loosening states than previous detecting technologies developed so far (acoustic, vibrometric, magnetic induction, deformation and bioelectric impedance) [19]. The use of hydroxyapatite-based layers provided additional detection sensibility (Figure 14), demonstrating the feasibility of using a cosurface capacitive sensing system to design effective multifunctional smart implants. Moreover, additional findings also highlight the ability of this sensing system to also be used as a therapeutic stimulation system: a higher detection sensibility was achieved for an excitation frequency (32 kHz) around the ones in which significant peri-implant bone regeneration occur (Figure 14), also ensuring higher average capacitance variations from weak to strong bonding scenarios. The developed web page (Figure 4) allows both clinicians and patients to evaluate the bone–implant fixation states on a personalized basis. Furthermore, clinicians can also control the operation of the capacitive sensing network using the Master Structure for data acquisition. This evaluation can be performed according to personalized periodicities (even daily if a tight follow-up is required) without demanding additional medical appointments and medical testing using imaging technologies. In contrast to the technologies already developed, striped and interdigitated capacitive architectures can be manufactured to provide flexible integration of sensing systems inside the instrumented implants and according to different geometries of implant surfaces. The sensing network also allows target-oriented: (i) monitoring of peri-implant regions: (ii) monitoring of different biointerfaces and their fixation states. The developed web server hosted on Raspberry Pi allows continuous monitoring of patients during their quotidian life. Furthermore, this sensing system can detect micro-scale and macro-scale bonding, debonding or early loosening states. Another important feature of these capacitive systems is related to their electrical power consumption: they require very low electric currents and voltages lower than 5 V, which enables the replacement of conventional power systems (batteries) by energy harvesting technologies that convert body motion into energy [47,48,49]. Adopting these technologies instead of batteries: (i) enables the overall monitoring system integration inside the implant; (ii) allows a longer lifetime, once there is no need to replace the power supply.

Future research must be carried out to assess the impact of different fibrous tissues and varying temperatures in peri-implant regions. As multifunctional smart implants aim for long-time survival, algorithms must be designed to address the impact of time-dependent bone quality. The sensing performance must be deeply analyzed using more realistic bone–implant interface states, which will require the development of a miniaturized circuit comprising multiple capacitance-to-digital converters. In vivo-implantation of these sensing systems will require the use of biocompatible, non-cytotoxic electrodes materials such as self-doped sulfonated polyaniline (SPAN)-based electrodes [50].

It is also important that future web applications can provide additional data related to biomechanical quantities, such as forces, moments, deformations and temperatures. Finally, clinical validation is required to provide undoubted proof of the effectiveness of this capacitive sensing.

## Data Availability

The data that support the findings of this study are available from the corresponding author upon request.

## Abbreviations

The following abbreviations are used in this manuscript:

PCB	Printed Circuit Board
SEM	Scanning Electron Microscope scanning
UML	Unified Modeling Language

## References

[1] March L., Smith E.U.R., Hoy D.G., Cross M.J., Sanchez-riera L., Blyth F., Buchbinder R., Vos T., Woolf A.D. (2014). Best Practice & Research Clinical Rheumatology Burden of disability due to musculoskeletal (MSK) disorders. Best Pract Res. Clin. Rheumatol..

[2] Kassebaum N.J., Arora (2016). Global, regional, and national disability-adjusted life-years (DALYs) for 315 diseases and injuries and healthy life expectancy (HALE), 1990–2015: A systematic analysis for the Global Burden of Disease Study 2015. Lancet.

[3] Anandacoomarasamy A., Fransen M., March L. (2009). Obesity and the musculoskeletal system. Curr. Opin. Rheumatol..

[4] Ferguson R.J., Palmer A.J., Taylor A., Porter M.L., Malchau H., Glyn-Jones S. (2018). Hip replacement. Lancet.

[5] Labek G., Thaler M., Janda W., Agreiter M., Stöckl B., Surgeon O. (2011). Revision rates after total joint replacement cumulative results from worldwide joint register datasets. Bone Joint J..

[6] Carr A.J., Robertsson O., Graves S., Price A.J., Arden N.K., Judge A., Beard D.J. (2012). Knee replacement. Lancet.

[7] Soares dos Santos M.P., Bernardo R., Henriques L., Ramos A., Ferreira J.A.F., Furlani E.P., Torres Marques A., Simões J.A.O. (2021). Towards an effective sensing technology to monitor micro-scale interface loosening of bioelectronic implants. Sci. Rep..

[8] Sumner D.R. (2015). Long-term implant fi xation and stress-shielding in total hip replacement. J. Biomech..

[9] Pivec R., Johnson A.J., Mears S.C., Mont M.A. (2012). Hip arthroplasty. Lancet.

[10] Soares dos Santos M.P., Ferreira J.A., Ramos A., Simões J.A., Morais R., Silva N.M., Santos P.M., Reis M.J., Oliveira T. (2013). Instrumented hip implants: Electric supply systems. J. Biomech..

[11] Standring S., Borley N.R., Gray H. (2008). Gray’s Anatomy: The Anatomical Basis of Clinical Practice.

[12] Nordin M. (2012). ; H. Frankel, V. Basic Biomechanics of the Musculoskeletal System.

[13] Kattimani V.S., Kondaka S., Lingamaneni K.P. (2016). Hydroxyapatite—Past, Present, and Future in Bone Regeneration. Bone Tissue Regen. Insights.

[14] Liu Y., Rath B., Tingart M., Eschweiler J. (2020). Role of implants surface modification in osseointegration: A systematic review. J. Biomed. Mater. Res..

[15] Goodman S.B., Yao Z., Keeney M., Yang F. (2013). The future of biologic coatings for orthopaedic implants. Biomaterials.

[16] Dos Santos M.P., Marote A., Santos T., Torrão J., Ramos A., Simões J.A., Da Cruz E Silva O.A., Furlani E.P., Vieira S.I., Ferreira J.A. (2016). New cosurface capacitive stimulators for the development of active osseointegrative implantable devices. Sci. Rep..

[17] Soares Dos Santos M.P., Ferreira J.A., Ramos A., Simões J.A. (2015). Active orthopaedic implants: Towards optimality. J. Franklin. Inst..

[18] De Sousa B.M., Correia C.R., Ferreira J.A., Mano J.F., Furlani E.P., Soares dos Santos M.P., Vieira S.I. (2021). Capacitive interdigitated system of high osteoinductive/conductive performance for personalized acting-sensing implants. NPJ Regen. Med..

[19] Cachão J.H., Dos Santos M.P., Bernardo R., Ramos A., Bader R., Ferreira J.A., Marques A.T., Simões J.A. (2020). Altering the course of technologies to monitor loosening states of endoprosthetic implants. Sensors.

[20] Soares dos Santos M.P., Coutinho J., Marote A., Sousa B., Ramos A., Ferreira J.A., Bernardo R., Rodrigues A., Marques A.T., Cruz e Silva O.A. (2019). Capacitive technologies for highly controlled and personalized electrical stimulation by implantable biomedical systems. Sci. Rep..

[21] Singh A.V., Dad Ansari M.H., Dayan C.B., Giltinan J., Wang S., Yu Y., Kishore V., Laux P., Luch A., Sitti M. (2019). Multifunctional magnetic hairbot for untethered osteogenesis, ultrasound contrast imaging and drug delivery. Biomaterials.

[22] Unger A.C., Cabrera-Palacios H., Schulz A.P., Jürgens C., Paech A. (2009). Acoustic monitoring (RFM) of total hip arthroplasty results of a cadaver study. Eur. J. Med. Res..

[23] Alshuhri A.A., Holsgrove T.P., Miles A.W., Cunningham J.L. (2015). Development of a non-invasive diagnostic technique for acetabular component loosening in total hip replacements. Med. Eng. Phys..

[24] Alshuhri A.A., Holsgrove T.P., Miles A.W., Cunningham J.L. (2017). Non-invasive vibrometry-based diagnostic detection of acetabular cup loosening in total hip replacement (THR). Med. Eng. Phys..

[25] Goossens Q., Leuridan S., Henyš P., Roosen J., Pastrav L., Mulier M., Desmet W., Denis K., Vander Sloten J. (2017). Development of an acoustic measurement protocol to monitor acetabular implant fixation in cementless total hip Arthroplasty: A preliminary study. Med. Eng. Phys..

[26] Glaser D., Komistek R.D., Cates H.E., Mahfouz M.R. (2008). Clicking and squeaking: In vivo correlation of sound and separation for different bearing surfaces. J. Bone Joint Surg.

[27] Ewald H., Timm U., Bader R., Kluess D. Acoustic sensor system for loosening detection of hip implants. Proceedings of the 2011 IEEE SENSORS.

[28] Georgiou A.P., Cunningham J.L. (2001). Accurate diagnosis of hip prosthesis loosening using a vibrational technique. Clin. Biomech..

[29] Rieger J.S., Jaeger S., Schuld C., Kretzer J.P., Bitsch R.G. (2013). A vibrational technique for diagnosing loosened total hip endoprostheses: An experimental sawbone study. Med. Eng. Phys..

[30] Rieger J.S., Jaeger S., Kretzer J.P., Rupp R., Bitsch R.G. (2015). Loosening detection of the femoral component of hip prostheses with extracorporeal shockwaves: A pilot study. Med. Eng. Phys..

[31] Lannocca M., Varini E., Cappello A., Cristofolini L., Bialoblocka E. (2007). Intra-operative evaluation of cementless hip implant stability: A prototype device based on vibration analysis. Med. Eng. Phys..

[32] Pastrav L.C., Jaecques S.V., Jonkers I., Perre G.V.D., Mulier M. (2009). In vivo evaluation of a vibration analysis technique for the per-operative monitoring of the fixation of hip prostheses. J. Orthop. Surg. Res..

[33] Jiang C.C., Lee J.H., Yuan T.T. (2000). Vibration arthrometry in thé patients with failed total knee replacement. IEEE Trans. Biomed. Eng..

[34] Ruther C., Nierath H., Ewald H., Cunningham J.L., Mittelmeier W., Bader R., Kluess D. (2013). Investigation of an acoustic-mechanical method to detect implant loosening. Med. Eng. Phys..

[35] Ewald H., Ruther C., Mittelmeier W., Bader R., Kluess D. A novel in vivo sensor for loosening diagnostics in total hip replacement. Proceedings of the 2011 IEEE SENSORS Proceedings.

[36] Burton A.R., Sun P., Lynch J.P. (2019). Bio-compatible wireless inductive thin-film strain sensor for monitoring the growth and strain response of bone in osseointegrated prostheses. Struct. Health Monit..

[37] McGilvray K.C., Unal E., Troyer K.L., Santoni B.G., Palmer R.H., Easley J.T., Demir H.V., Puttlitz C.M. (2015). Implantable microelectromechanical sensors for diagnostic monitoring and post-surgical prediction of bone fracture healing. J. Orthop. Res..

[38] Arpaia P., Clemente F., Zanesco A. (2007). Low-invasive diagnosis of metallic prosthesis osseointegration by electrical impedance spectroscopy. IEEE Trans. Instrum. Meas..

[39] Arpaia P., Clemente F., Romanucci C. In-vivo test procedure and instrument characterization for EIS-based diagnosis of prosthesis osseointegration. Proceedings of the Conference Record—IEEE Instrumentation and Measurement Technology Conference.

[40] Graichen F., Arnold R., Rohlmann A., Bergmann G. (2007). Implantable 9-channel telemetry system for in vivo load measurements with orthopedic implants. IEEE. Trans. Biomed. Eng..

[41] Morais R., Frias C.M., Silva N.M., Azevedo J.L., Serôdio C.A., Silva P.M., Ferreira J.A., Simões J.A., Reis M.C. (2009). An activation circuit for battery-powered biomedical implantable systems. Sens. Actuator A Phys..

[42] Harun W.S., Asri R.I., Alias J., Zulkifli F.H., Kadirgama K., Ghani S.A., Shariffuddin J.H. (2018). A comprehensive review of hydroxyapatite-based coatings adhesion on metallic biomaterials. Ceram. Int..

[43] Herrera A., Mateo J., Gil-Albarova J., Lobo-Escolar A., Ibarz E., Gabarre S., Más Y., Gracia L. (2015). Cementless hydroxyapatite coated hip prostheses. Biomed Res. Int..

[44] Singh A.V., Maharjan R.S., Kromer C., Laux P., Luch A., Vats T., Chandrasekar V., Dakua S.P., Park B.W. (2021). Advances in Smoking Related in Vitro Inhalation Toxicology: A Perspective Case of Challenges and Opportunities from Progresses in Lung-on-Chip Technologies. Chem. Res. Toxicol..

[45] Shah F.A., Thomsen P., Palmquist A. (2019). Osseointegration and current interpretations of the bone-implant interface. Acta Biomater..

[46] Azami M., Moztarzadeh F., Tahriri M. (2010). Preparation, characterization and mechanical properties of controlled porous gelatin/hydroxyapatite nanocomposite through layer solvent casting combined with freeze-drying and lamination techniques. J. Porous Mater..

[47] Vidal J.V., Slabov V., Kholkin A.L., dos Santos M.P. (2021). Hybrid Triboelectric-Electromagnetic Nanogenerators for Mechanical Energy Harvesting: A Review.

[48] Carneiro P., Soares dos Santos M.P., Rodrigues A., Ferreira J.A., Simões J.A., Marques A.T., Kholkin A.L. (2020). Electromagnetic energy harvesting using magnetic levitation architectures: A review. Appl. Energy.

[49] Soares Dos Santos M.P., Ferreira J.A., Simões J.A., Pascoal R., Torrão J., Xue X., Furlani E.P. (2016). Magnetic levitation-based electromagnetic energy harvesting: A semi-Analytical non-linear model for energy transduction. Sci. Rep..

[50] Min Y., Liu Y., Poojari Y., Wu J.C., Hildreth B.E., Rosol T.J., Epstein A.J. (2014). Self-doped polyaniline-based interdigitated electrodes for electrical stimulation of osteoblast cell lines. Synth. Met..

